# Thin Hydrogel Films for Optical Biosensor Applications

**DOI:** 10.3390/membranes2010040

**Published:** 2012-02-08

**Authors:** Anca Mateescu, Yi Wang, Jakub Dostalek, Ulrich Jonas

**Affiliations:** 1 Foundation for Research and Technology—Hellas (FORTH), Institute of Electronic Structure & Laser (IESL), Bio-Organic Materials Chemistry Laboratory (BOMCLab), Nikolaou Plastira 100, Vassilika Vouton, Heraklion 71110, Crete, Greece; Email: mateescu@iesl.forth.gr; 2 AIT Austrian Institute of Technology GmbH, Muthgasse 11, Vienna 1190, Austria; Email: Yi.Wang.fl@ait.ac.at; 3 Macromolecular Chemistry, Department of Chemistry-Biology, University of Siegen, Siegen 57076, Germany

**Keywords:** surface-attached hydrogel films, responsive hydrogels, optical biosensors, surface plasmon resonance spectroscopy, optical waveguide mode spectroscopy, affinity sensing

## Abstract

Hydrogel materials consisting of water-swollen polymer networks exhibit a large number of specific properties highly attractive for a variety of optical biosensor applications. This properties profile embraces the aqueous swelling medium as the basis of biocompatibility, non-fouling behavior, and being not cell toxic, while providing high optical quality and transparency. The present review focuses on some of the most interesting aspects of surface-attached hydrogel films as active binding matrices in optical biosensors based on surface plasmon resonance and optical waveguide mode spectroscopy. In particular, the chemical nature, specific properties, and applications of such hydrogel surface architectures for highly sensitive affinity biosensors based on evanescent wave optics are discussed. The specific class of responsive hydrogel systems, which can change their physical state in response to externally applied stimuli, have found large interest as sophisticated materials that provide a complex behavior to hydrogel-based sensing devices.

## 1. Introduction

The general term “gel” is defined as a viscoelastic solid-like but deformable material composed of a dispersion of a substantially diluted network in a continuous gas or liquid medium. In the case of a gas medium the system is called either *aerogel* (highly porous) or *xerogel* (collapsed, dry), whereas a host for liquids is referred to as *lyogel*. In the case of *hydrogels*, the dispersing medium is water [1], and this material is the focus of the present review. The network-forming material comprises either small aggregating molecules [2], particles, or polymers that form extended elongated structures with interconnections (the crosslinks) between the segments. The crosslinks can be of either chemical nature, in the form of covalent bonds, or of physical nature in the form of coordinative, electrostatic, hydrophobic, dipole-dipole interactions or chain entanglements between the network segments. Polymers represent a rich class of materials that yield a large pallet of different gel types. In this review, the emphasis will be placed on polymer-based hydrogels, which are insoluble, cross-linked water-swollen polymer networks of hydrophilic homopolymers or copolymers.

Hydrogel materials possess many specific properties that make them attractive for a wide range of applications. Their ability to contain water, stability in aqueous media and softness, makes hydrogels compatible with biological systems and thus they were proposed for different biomedical applications such as tissue engineering, cell adhesion, wound healing, controlled drug release, contact lenses, to name a few [3,4,5,6,7,8,9,10,11]. Hydrophilic polymer layers were also employed as support for lipid bilayer membranes, in particular for electric measurements of transport phenomena involving membrane proteins [12,13,14,15,16].

Owing to the highly open structure and large inner surface, hydrogels can accommodate large amounts of molecules with specific functions and are becoming irreplaceable materials in biosensing technology for detection of chemical or biological analytes [17,18,19,20,21,22]. In many biosensor applications, hydrogel materials are employed at an interface between the to-be-analyzed aqueous sample fluid and the sensor signal transducer. Typically, the hydrogel interface is modified with biomolecular recognition elements (BREs) such as antibodies, enzymes or with biomimetic molecular imprinted moieties in order to selectively recognize specific target analytes. Compared to other types of biointerfaces (e.g., based on 2D self-assembled monolayers—SAMs), the 3D nature of the hydrogel networks allows to accommodate orders of magnitude larger amounts of recognition elements [23], provides a more natural microenvironment for biomolecules that increases their stability [24] and offers routes to implement additional functionalities (e.g., separation of target analyte from other molecules in a sample) [25]. In addition, the class of “smart” gels that respond to external stimuli become of increasing interest in biosensor research. For instance, miniature holographic diffraction elements based on these responsive hydrogel materials were integrated in a contact lens for the real-time monitoring of glucose levels in an artificial tear fluid by a diffracting wavelength shift [26]. Moreover, appropriate selection of the polymer building blocks and introduction of appropriate functional groups allows tuning of the responsive behavior. The mechanical work that can be performed by the swelling and collapse process is exploited in actuation, which forms the basis for valves, pumps and potentially for micromachines. In the following, the chemical nature, particular properties and specific application in biosensing of surface-attached hydrogel films are discussed. 

## 2. Classification of Hydrogel Systems

**Figure 1:**
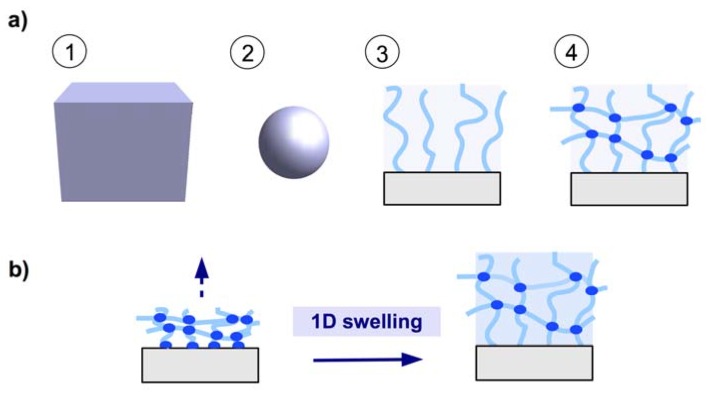
(**a**) Schematic representation of hydrogel formats (1) 3D bulk gel, (2) microgel particle, (3) brush layer, and (4) surface-attached polymer network. (**b**) Surface-attached polymer network swelling in one dimension away from the substrate.

There are four generic formats of a hydrogel material as depicted in Figure 1a: (1) the bulk material as a 3D hydrogel; (2) microgels as small particles; (3) brush layers with individual polymer chains attached onto a solid support; and (4) thin hydrogel network films, preferentially attached to a solid support (see Figure 1a). Hydrogel films combine the advantage of a robust network architecture of 3D bulk gels and microgel particles with the high surface-to-volume ratio of microgels and brush layers. By anchoring the polymer network to a solid substrate at multiple points, the mechanical stability of the thin gel layers is greatly increased. The swelling behavior is mainly determined by the balance between the expanding force (induced by the osmotic pressure of polymer solvation) and the restoring force (of the chain segments between the crosslinks). Because the polymer network is anchored onto the substrate, a quasi one-dimensional swelling in the direction away from the substrate occurs, as illustrated in Figure 1b. 

Hydrogel materials can be further classified according to their origin, their chemical structure, or their properties. The two fundamental sources for hydrogel materials are nature and synthesis. Naturally occurring hydrogels can be of abiotic origin (or a product of biological processes in living organisms, while synthetic hydrogels in the context of this review refer to a polymer backbone of organic chemical nature based on carbon atoms. Classification according to the chemical structure gives a large variety of hydrogel types and allows a systematic distinction with respect to chemical properties and reactivity. The material properties such as responsiveness to external stimuli, biodegradability, biocompatibility, or non-fouling behavior form another basis for classification.

### 2.1. Chemical Structures of Polymer-Based Hydrogels

A chemical classification can be based on the molecular structure of the individual subunits (the repeating- or monomer units) that compose the polymer of the hydrogel material and on the type of linkage between these units. In order to attract water during the swelling process of the hydrogel, these subunits must be intrinsically hydrophilic or contain enough hydrophilic side groups such as hydroxyl-, amide- or carboxyl moieties.

#### 2.1.1. Main Monomer

The main monomer is the dominant building block of the polymer structure and can differ substantially, particularly between hydrogels of biological and synthetic origin. Typical polymers of biological origin are polypeptides, polynucleotides and polysaccharides, such as agarose, alginate, chitin, chitosan, cellulose, dextran, or hyaluronic acid. These naturally occurring polymers exhibit biocompatible and biodegradable properties and can be chemically modified to introduce the required functionalities for network formation. In synthetic hydrogels a large fraction is based on vinyl derivatives with olefinic double bonds that can undergo radical polymerization to form the polymer backbone. Some characteristic examples are poly([meth/]acrylic acid) (this spelling meaning poly(acrylic acid) or poly(methacrylic acid)), poly[meth/]acrylates, poly[meth/]acrylamides, poly(vinyl alcohol), polyvinylpyrrolidone and their corresponding copolymers. Other examples of synthetic polymer types for hydrogels are polyethers, polyurethanes, and poly(ethylene glycol). 

##### 2.1.1.1 Monomers and Polymers for Hydrogel Films

In the examples below, natural and synthetic polymer systems relevant to hydrogel films are presented. Natural-based polymers such as dextran hydrogel films have been immobilized on aminated poly(ethylene terephthalate) substrates by photochemical immobilization and concurrent crosslinking of a 4-azidobenzoic acid-modified dextran [27]. Similarly, benzophenone-based dextran hydrogel layers were attached onto benzophenone-functionalized gold substrates [23]. Such crosslinked dextran films possess protein- and cell-repelling properties and render the substrate surface, onto which they are tethered, highly hydrophilic and non-fouling. In another study, polyamide 6,6 fabrics were modified with chitosan-based hydrogels by crosslinking the primary amine groups of chitosan with terminal amine groups of the polyamide and the moisture absorption capacity of the modified fabrics was investigated [28].

A synthetic biocompatible hydrogel surface was demonstrated by a polymer film composed of a network with polymethacrylate backbone with pendant zwitterionic phosphorylcholine groups, lauryl chains, and 2-hydroxypropyl groups, crosslinked via trimethoxysilane moieties attached to the backbone [29]. Synthetic polymers that are frequently used in hydrogel layer systems are acrylate derivatives such as [meth/]acrylamides. Their structure and properties can be effectively tailored by substitution of the amide protons with side chains, which leads to a large number of hydrogel systems with specific behavior and functions. For example, the different swelling behavior between unrestricted 3D gels and thin, surface-attached films was investigated by ellipsometry in photocrosslinked poly(dimethylacrylamide) hydrogels [30]. The overall swelling of the film was found to be reduced compared to the bulk material, while the 1D layer swelling was larger than expected from simple geometrical considerations. A microcantilever could be successfully modified with a polyacrylamide hydrogel film, which had the ionic comonomer (3-acrylamidopropyl)trimethylammonium chloride incorporated for potential applications in sensors [31]. The cationic group in the comonomer would bind CrO_4_^2−^ ions from the aqueous environment, which in turn changed the swelling state of the hydrogel and therefore the cantilever deflection. This deflection change was detected and used as a measure of the ion concentration. In another study, indium-tin oxide (ITO) surfaces were modified with a thin hydrogel layer based on poly(*N*-(2-hydroxyethyl)acrylamide) and employed as a soft “cushion” to form a protein-supported bilayer lipid membrane. The hydrogel films with mesh sizes smaller than the size of the protein were provided with a nitrilotriacetic acid functionality, which chelates with Ni^+^ ions designed to bind the cytochrome c oxidase protein in a well-defined orientation. Since the protein is too big to penetrate the gel, it remained on the surface and the lipid bilayer was formed around the protein on the hydrogel top [32].

Alkyl-substituted acrylamides form polymers with an interesting temperature-dependent solubility in water. These polymers easily dissolve at low temperatures, while raising the temperature above the so-called lower critical solution temperature (LCST) leads to precipitation from the aqueous phase. Such alkylated acrylamides form the basis of so-called responsive hydrogels, which can abruptly change their macroscopic swelling state upon passing the LCST [33]. An example is *N*-isopropylacrylamide with the corresponding homopolymer showing a LCST of about 32 °C. This monomer has been used in many hydrogel film systems, frequently in combination with other comonomers that can alter polarity, charge, and facilitate crosslinking [34,35,36,37,38,39,40,41,42,43,44,45,46,47,48]. The phase transition temperature in thin poly(*N*-isopropylacrylamide) hydrogel films can be varied by addition of other comonomers. For example, the LCST was varied from 25 °C for a poly(2-vinylpyridine)-poly(*N*-isopropylacrylamide) block copolymer to 43 °C for a poly(*N*,*N-*dimethylacrylamide-*co*-*N*-isopropylacrylamide) copolymer [37]. Another example of thermoresponsive hydrogel type is based on polyvinyl derivatives. This was shown for poly(*N*-vinylcaprolactam) hydrogel films immobilized on track-etched poly(ethylene terephthalate) membranes for temperature-dependent separation of dextrans of different molecular weight [49].

#### 2.1.2. Functional Groups

In hydrogel networks, the functional groups are molecular units of the polymer chains that posses specific properties to allow further chemical modification of the hydrogel or afford the network with additional functional properties. Some examples of functional groups are carboxylic acid-, amino-, and hydroxyl moieties, which are either intrinsically part of the polymer or can be subsequently introduced into the hydrogel after network formation. The functional groups can influence the hydrogel behavior, like swelling characteristics, the lower or upper critical solution temperature in responsive gels, the charge and that also allow post-modification of the hydrogel network, for example to introduce biologically active ligands. Here, a range of functional groups have been employed, which can undergo efficient coupling reactions by the so-called click reactions under mild conditions [14]. An example of functional groups, which control the swelling behavior, are the carboxylic acid groups of poly(methacrylic acid) or poly(acrylic acid) in pH sensitive hydrogel layers [50,51,52]. By increasing the pH, the carboxyl groups are charged and the hydrogel layer swells, which can be detected in an appropriate sensor setup. The carboxyl groups can be further activated to bind biologically active species, such as antibodies or fluorescence labels for applications in surface plasmon resonance (SPR) and optical waveguide sensors. For instance, the carboxyl groups in crosslinked carboxymethyl dextran brush layers were activated with 1-ethyl-3-(3-dimethylaminopropyl)carbodiimide and *N*-hydroxysuccinimide, and reacted with a fluorescence dye [53]. The modification of the layer swelling state due to the variation of the salt concentration or pH were recorded as a measure of the changes in the fluorescence signal from the dye-functionalized dextran. In another example, immunoglobulin G (IgG) antibodies were covalently immobilized in poly(*N*-isopropylacrylamide) hydrogel layers containing carboxyl groups by reaction via 1-ethyl-3-(3-dimethylaminopropyl)carbodiimide, *N*-hydroxysuccinimide, and sodium *para-*tetrafluorophenol sulfonate to yield responsive sensor matrices for specific immuno-binding [54,55]. In a similar way, IgG antibodies were immobilized in a hydrogel layer of photocrosslinkable carboxymethylated dextran for the detection of the free prostate-specific antigen by employing a biosensor platform based on surface plasmon-enhanced fluorescence spectroscopy with long-range surface plasmons [23]. Furthermore, functional groups may be incorporated in the hydrogel-forming polymer in a protected form and deprotected in the hydrogel after network formation. In this context, the utilization of photosensitive protecting groups provides the possibility to chemically pattern the hydrogel film by site-selective deprotection of the functional groups through masked irradiation in a photolithographic manner. Such a procedure was illustrated in a chemically crosslinked hydrogel film based on polyacrylamide with the amino groups being protected by photoactive *o*-nitrobenzyl carbamates [56]. Photolithographic illumination of the hydrogel generated a pattern with free amino groups in the irradiated regions accessible for further chemical modification.

#### 2.1.3. Crosslinkers

**Figure 2:**
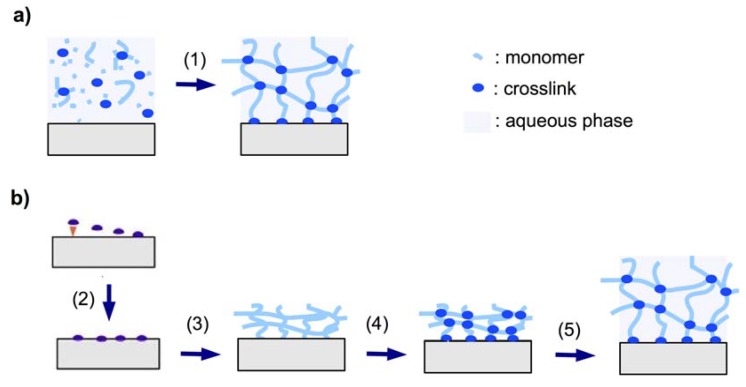
Schematic representation of (**a**) *in situ* crosslinking polymerization (1) and surface attachment of the hydrogel in the swollen state, and (**b**) post-synthetic crosslinking by first immobilizing an adhesion-promoting molecule (2), followed by deposition of a prepolymer (3), crosslinking of the dry layer (4), and subsequent swelling of the gel (5).

The crosslinker is defined as the molecular entity at which three or more chains can join to form a crosslink point and turn the polymer into a network. There are two different methods that can be employed to create the crosslink points. In the so-called “ *in situ* crosslinking” method the crosslink is created during the synthesis of the polymer chains by introducing the crosslinker molecules (Figure 2a). Alternatively, in “post-synthetic crosslinking”, the crosslink points may be generated in a second step after formation of the polymer chains (Figure 2b).

##### 2.1.3.1. *In Situ* Crosslinking

The *in situ* crosslinking method relies on the formation of the polymer chain in the presence of a crosslinking monomer with two or more polymerizable sites (see Figure 2a). For instance, photo- and thermally induced *in situ* polymerization of acrylamide and simultaneous crosslinking by *N,N’*-methylenebisacrylamide in aqueous solution was employed for the preparation of hydrogel films with embedded silicon pillar arrays for application in microactuation by drying and swelling [57]. Similarly, thin hydrogel films were prepared on sensor cantilevers for the detection of chromic acid anions [31]. In another study, pH sensitive hydrogel layers were attached onto microcantilevers by bulk photopolymerization of methacrylic acid and poly(ethylene glycol) dimethacrylate as the crosslinker moiety. Upon base-induced swelling of the hydrogel, a pH increase was measured by the deflection of the cantilever [50]. Bis- and tetra-acrylated poly(ethylene glycol) derivatives were also used in a lithographic photopolymerization process. The highly crosslinked hydrogel film structures, which were generated, were used as supports for protein immobilization [58]. An alternative to crosslinking polymerization in solution or bulk is plasma polymerization. Plasma polymerization processes induce crosslinking besides polymerization, even in the absence of a crosslinker molecule due to the strong ionization conditions and molecular fragmentation [59]. This process was illustrated by vapor-phase deposition of poly(*N*-isopropylacrylamide) hydrogel films [60].

##### 2.1.3.2. Post-Synthetic Crosslinking

**Figure 3:**
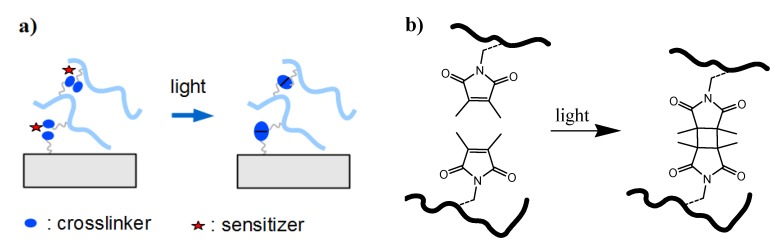
(**a**) Schematic representation of a dimerization process requiring two reactive units per crosslink point and potentially a sensitizer molecule. (**b**) Example of a [2+2] photo-cyclodimerization reaction with two dimethymaleimide moieties.

In the post-synthetic crosslinking method, the polymer film is firstly formed on a substrate from a soluble precursor polymer, followed by crosslinking in a second step (see Figure 2b). The crosslinking process may involve exposure to chemical agents, elevated temperatures, or radiation of visible or UV light, high-energy photons, electrons, or ions, and often induces simultaneous covalent attachment of the polymer film to the substrate. Photocrosslinking is a convenient procedure that requires only relatively simple instrumentation without the use of specific chemicals. The photon energy and dose can be adjusted by the light source, the irradiation intensity, and the exposure time, allowing large control over the crosslink density of the polymer network. Various comonomers have been incorporated as photocrosslinking units into the polymer chains of hydrogel precursor materials. For examples, a [2+2] cyclodimerization of 2-(dimethylmaleimido)-*N*-ethylacrylamide was used in the presence of the photosensitizer thioxanthone for the photocrosslinking of poly(*N*-isopropylacrylamide) hydrogel films (see Figure 3a and b) [44]. In this reaction, a cyclobutane ring is formed as crosslink by the dimerization of two maleimido double bonds under UV irradiation. The crosslink density can be adjusted by the concentration of the crosslinker comonomer in the polymer as well as by the irradiation time and has a direct influence on the mechanical properties and the thermal response of the hydrogel film [41]. By using masked illumination, thermoresponsive hydrogel films could also be lithographically patterned on the substrate [45].

**Figure 4:**
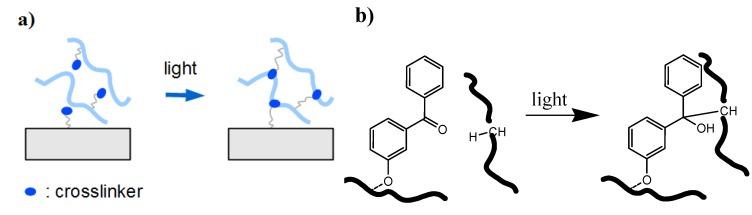
(**a**) Schematic representation of an insertion process involving only one photoreactive molecule. (**b**) Example of a photoinduced C-H insertion with one benzophenone molecule.

The benzophenone group represents another very powerful photocrosslinker unit. Upon irradiation with UV light, the benzophenone group undergoes an nπ* transition to the triplet biradical of the carbonyl bond inserting into any available non-aromatic C-H bond and forming a covalent crosslink (see Figure 4a and b). A benzophenone-based comonomer such as methacryloyloxybenzophenone has been incorporated into poly(*N*,*N*-dimethylacrylamide) or poly(*N*-isopropylacrylamide) for the preparation of photocrosslinked hydrogel films [30,35]. The advantages of such benzophenone crosslinkers are: (i) the ability to undergo photocrosslinking without the need of a sensitizer moiety; (ii) that only one individual benzophenone molecule is required for the crosslinking reaction; and (iii) that the C-H insertion reaction is unspecific, allowing the photocrosslinking of various polymer types. 

Azidophenyl derivatives with an N_3_-group at a phenyl ring provide the same advantages as a photocrosslinker unit as benzophenones do. The only disadvantage is their lower thermal stability and higher reactivity. The azido group fragments upon irradiation with light into N_2_ and a phenyl-bound nitrene, which itself undergoes rearrangements and insertion reactions into neighboring polymer chains. This azidophenyl crosslinker has been incorporated into poly(*N*-isopropylacrylamide), dextrans and poly(*N*-vinylcaprolactam) polymers for hydrogel film preparation [27,49,61]. The chemically related phenylsulfonyl azide group is thermally reactive and was incorporated as styrenesulfonyl azide into various polymers for hydrogel formation. This sulfonyl azide comonomer leads to polymer network formation upon heating of the polymer film. It also allows topographic structuring by hot embossing of the polymer film under concurrent crosslinking [62].

Thermally induced crosslinking of thin hydrogel films was also illustrated for a polymer blend of poly(acrylic acid)/poly(vinyl alcohol). The thermal crosslinking induces ester formation between the carboxylic acid on one polymer chain and the hydroxyl group on the other chain [51,52]. Chemical crosslinking with a crosslinking agent, which is added to the polymer layer after the film formation, was reported for a large number of hydrogel systems and corresponding reactive agents. One example is the crosslinking of carboxymethylated dextran brush layers by the addition of bisamino-crosslinking moieties after activation of the carboxylic acid groups in the dextran brush [53]. Besides diamines, polyfunctional amines in the form of amino-functionalized silica nanoparticles were employed as crosslinking agents. The amino function of the silica particles reacted with the acryloxysuccinimide groups of a poly(*N*-isopropylacrylamide-*co*-*N*-acryloxysuccinimide) film to form amide bonds [46]. In another study, chemical crosslinking was achieved for a copolymer of *N*-*tert*-butylacrylamide and acrylamide by addition of 1,2,3,4-butanetertracarboxylic acid as crosslinker and sodium hypophosphite as catalyst [63]. More sophisticated crosslinking reactions have been reported with efficient high-yield reactions (“click chemistry”), which can be performed under mild conditions in the presence of other functional groups [14].

### 2.2. Surface Attachment Strategies

Stable attachment of hydrogel layers onto a solid substrate is of major importance for many applications, such as coatings and sensing layers. Depending on the chemical nature of the hydrogel polymer and the substrate material, specific surface bonding strategies have been developed. Adhesion-promoting molecules are often employed in order to facilitate the bonding of the hydrogel with the substrate. Such intermediate molecular layers interact strongly with the substrate material via an anchor group, while the other part of these molecules bonds with the hydrogel polymer. For instance thiol anchor groups are often used on gold surfaces, while alkylalkoxysilanes are successfully employed on oxidic substrates like glass. In the case of polymeric substrates, the surfaces are often pretreated by exposure to harsh reactive conditions (like plasma discharges) to generate functional groups in the polymer substrate followed by covalent attachment of the hydrogel or the adhesion promoter layer to mediate the hydrogel binding.

**Figure 5:**
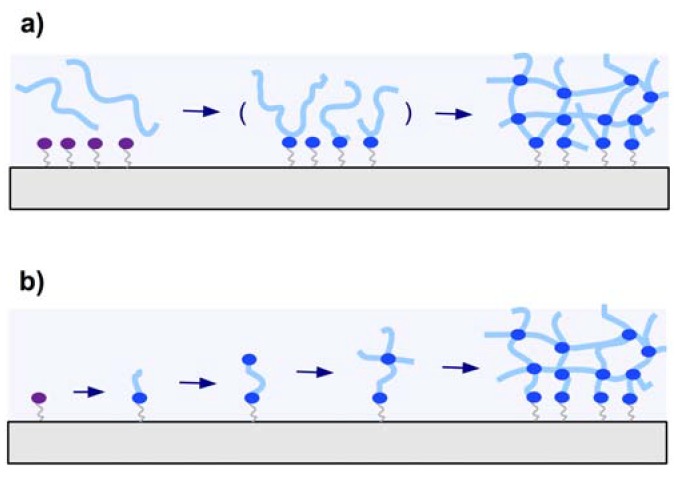
Schematic representation of (**a**) the “grafting to” and (**b**) the “grafting from” techniques.

There are two general methods employed for attaching hydrogel polymers on surfaces. In Figure 5a the “*grafting to*” technique is depicted, where a preformed polymer chain is attached to the substrate. The other method is the “*grafting from*” technique illustrated in Figure 5b, which involves the growing of the polymer chain from the substrate.

#### 2.2.1. “Grafting to” Method

In the “grafting to” technique, a preformed polymer chain is attachment to a substrate either by a covalent bond or by physical means via attractive interactions of the adsorbed polymer with the substrate. Grafting of single polymer chains to a substrate surface leads to polymer brush layers with low grafting densities and thicknesses roughly in the dimension of the polymer coil diameter. When the “grafting to” method is combined with interchain crosslinking, thick polymer networks can be formed from many polymer chains and simultaneously attached to the substrate (see Figure 5a).

##### 2.2.1.1. Chemical “Grafting to”

The chemical “grafting to” method employs a preformed polymer chain that is covalently bound to a substrate surface by a chemical reaction between a functional group on the polymer backbone and on the substrate. If the substrate carries appropriate functional groups, reactive moieties in the polymer can form directly covalent bonds without a need for surface modification. For instance, in cellulose fibers with surface hydroxyl groups a copolymer of *N*-*tert*-butylacrylamide and acrylamide was coupled with 1,2,3,4-butanetetracaboxylic acid as coupling and crosslinking agent [63]. There are cases where the polymer contains very reactive groups, which can be activated by either heat or light. Such activated species can react even with an organic substrate surface that does not carry specific functional groups. For example, heat activated surface attachment (usually in concurrence with crosslinking) was employed for phenylsulfonyl azide-containing polymers on an alkane surface layer formed by octadecyltrichlorosilane [62]. By insertion of the nitrene species (from the thermal decomposition of the azide) into a C-H bond of the alkyl chain a chemical bonding was formed.

If the substrate material does not carry appropriate functional groups for covalent bonding with the polymer network of the hydrogel, adhesion promoter layers that bind strongly to the substrate as well as to the polymer chains can be used. This strategy was used on silica surfaces coated with poly(glycidyl methacrylate-*co*-acrylic acid) as the reactive adhesion layer, onto which a chemically crosslinked polyacrylamide gel was deposited [57]. In another example, a wet-chemically oxidized polyethylene substrate was activated with ethyl chloroformate and subsequently reacted with branched polyethylenimine to yield surface amine groups, followed by chemisorption of a copolymer of *N*-isopropylacrylamide and *N*-acryloxysuccinimide [46]. The same type of polymer was immobilized in a similar way onto the amino surface of a self-assembled monolayer of 4-aminobutylmethoxydimethylsilane on glass and oxidized silicon substrates and employed as a support for neuronal cells growth [64].

##### 2.2.1.2. Photochemical “Grafting to”

There are two general strategies for the photochemical grafting of polymer chains onto substrates [65]:

(1) Bond formation between photoactivated functional groups of the polymer chain with the substrate material, which usually also leads to crosslinking of the polymer film.
(2) Reaction of photoreactive groups found at the substrate surface (e.g., from adhesion promotor layers) with the polymer chains of the hydrogel network.

A common photoreactive group that has been incorporated into polymer chains and used for surface-grafting of hydrogel layers to solid substrates is the phenyl azide moiety. Upon irradiation with light this group inserts into C-H bonds at the substrate surface. Some examples are poly(*N*-isopropylacrylamide) on polystyrene substrates [61], poly(*N-*vinylcaprolactam) on track-etched poly(ethylene terephthalate) membranes [49], and dextran on amino-functionalized poly(ethylene terephthalate) [27]. As already mentioned, the benzophenone unit is another photoactive group that can insert into C-H bonds upon irradiation with light. This moiety has been incorporated into the polymer chain as well as into adhesion promotor layers at the substrate surface to immobilize poly(*N*-isopropylacrylamide) and other polymers [35,66]. A more selective photoreaction was demonstrated with the 3,4-dimethylmaleimide group, which requires two of these moieties to participate in a light-induced dimerization to a cyclobutane product. As a consequence, the 3,4-dimethylmaleimide group had to be incorporated into the adhesion promoter as well as into the polymer chain [37,45].

#### 2.2.2. “Grafting from” Method

The general concept behind the “grafting from” technique is based on the polymer chain growth starting from an initiator site at the substrate surface. By the incorporation of monomer molecules from the surrounding medium, polymer brush layers with typically low film thicknesses limited by the chain length have been prepared [67,68]. If concurrent or subsequent crosslinking takes place during polymer growth, polymer network films are formed (see Figure 5b). Employing this technique, crosslinked poly(*N*-isopropylacrylamide) hydrogel layers with carboxyl groups were prepared on aminopropylsilane-modified silica particles, which were firstly reacted with 4,4’-azobis(4-cyanovaleric acid) as radical initiator via carbodiimide coupling [69]. Similarly, this azo-initiator was activated by irradiation with UV light to grow crosslinked poly(*N*-isopropylacrylamide) hydrogel films on silica coated gold layers [38]. Polyacrylamide-based hydrogels were covalently bonded to a polypropylene substrate modified with a functional polyperoxide, which initiates radical polymerization upon decomposition of the peroxide groups. A grafted hydrogel network was prepared in the presence of *N,N*’-methylene-bis-acrylamide as a cross-linking agent and potassium persulfate as an additional initiator in bulk [70]. Another type of photo-initiator is derived from sodium *N,N*-diethyldithiocarbamate, which was bound to glass surfaces and used to initiate the growth of crosslinked poly(*N*-isopropylacrylamide) films by irradiation with UV light [71].

A more common variation of the “grafting from” method for the synthesis of surface-attached hydrogel films employs adhesion promotor molecules at the substrate surface. These moieties carry a polymerizable group such as a vinylic double bond. In this case, the grafting polymerization is carried out on top of the substrate in a film of the monomers either neat or in solution with the free initiator mixed in. The substrate-anchored groups of the adhesion promotor take part in the polymerization process and get incorporated into the polymer chain, through which the network is covalently attached to the substrate. Using this method, surface-attached hydrogel films and microstructures were prepared from acrylated poly(ethylene glycol) on a 3-methacryloxypropylsilane-functionalized substrate [58], or from *N*-isopropylacrylamide on a 3-methacryloxypropylsilane layer [38,64,72], and from acrylamide on an allytriethoxysilane layer [31]. Surface-grafting polymerization was also performed from a neat polypropylene substrate that intrinsically carried active species after irradiation in an electron accelerator [73]. On this activated substrate, two interpenetrating polymers of polydimethylaminoethylmethacrylate and poly(acrylic acid) were grown successively from solution. Also, by employing electron beam irradiation, poly(*N*-isopropylacrylamide) layers were grown on polystyrene cell culture dishes [74]. 

### 2.3. Coating Methods

Two fundamental strategies for depositing hydrogels as coatings on solid surfaces can be distinguished: (i) deposition of the polymer layer followed by subsequent crosslinking; or (ii) crosslinking during deposition or growth of the polymer network. For the first method, the classical techniques of spin coating, film casting or doctor blading, spraying, and so on, are relevant. For example, film casting [49], dip casting [63], and spin coating [35,45,62] are widely used to first deposit a precursor polymer onto the solid substrate from solution, followed by crosslinking and surface attachment. In the second strategy, a solution of the monomers is deposited on the substrate, from which the polymerization and network formation process is initiated. Examples from literature are found in the above discussion of the “grafting from” method. The coating process can have a considerable influence on the properties of a hydrogel film made of the same polymeric material but using different coating methods. This could be explained by anisotropic chain conformation and orientation introduced during the coating process, for example through shear forces [41].

Various methods such as photolithographic structuring as well as hot embossing have been applied to laterally pattern the hydrogel films [33,75]. For example, if the polymerization is photoinitiated on a substrate possessing monomer functionalities, micropatterned hydrogel films can be obtained by irradiating the film of the monomer and crosslinker mixture through a photomask [58]. In a similar manner, if the crosslinking and surface attachment of a prepolymer film is mediated by light (e.g., by 3,4-dimethylmaleimide-, benzophenone-, or azido groups), masked illumination of the photoreactive film will produce a film pattern [45]. In the case where crosslinking and surface attachment are thermally initiated, for example by phenylsulfonyl azide groups, patterning can be induced by hot embossing of the uncrosslinked polymer film with a hot stamp [62].

## 3. Properties of Hydrogel Layers

In order to understand and control the properties of hydrogel layers, the morphological structure of the polymer networks that form the hydrogel is of prime importance. One generic feature of gels is the existence of inhomogeneities on a molecular level. These inhomogeneities result from clusters of higher polymer concentration that are interconnected with more dilute polymer chains [76]. Such a non-uniform spatiotemporal distribution of the chain and crosslink density in the gel network is caused by frozen concentration fluctuations, due to the permanent crosslinks, and by thermal concentration fluctuations of the dissolved polymer chains driven by Brownian motion [77]. From a molecular structural point of view, the inhomogeneities can be classified as:

- spatial inhomogeneities of non-uniform distribution of crosslinks in space,
- topological inhomogeneities of the network such as loops, trapped entanglements, and dangling chain ends,
- connectivity inhomogeneities of the polymer cluster distribution in size and space, which is also related to variations of the branching architecture of the network.

Up to now, various methods employed to study inhomogeneities of bulk gels have also been applied to surface-attached hydrogel layers. These include small-angle X-ray and neutron scattering [48], fluorescence correlation spectroscopy [78], and dynamic light scattering [79]. Furthermore, surface-layer-specific methods like ellipsometry [29,30,47], SPR [35,38,41], optical waveguide mode spectroscopy (OWS) [80], and atomic force microscopy (AFM) [39,41,81] were employed for the observation of hydrogel films.

Another characteristic of surface-attached hydrogel films is the swelling of the dry layer upon contact with water to an equilibrium film thickness [82]. The characteristic quantities related to this process are the swelling ratio or the swelling degree, which corresponds to the relative change of the film thickness, and the time evolution of the swelling process. Structural parameters that have a strong influence on the swelling ratio are the hydrophilicity of the polymer and the crosslinking density. For instance, more polar groups attract more water and, in particular, charged moieties with their counterions give rise to a large osmotic pressure and a strong drive for water molecules to hydrate the ions. This leads to an increased water uptake and larger swelling ratios [83,84]. As far as the crosslinking density is concerned, a higher crosslink density leads to a lower swelling ratio [30,44,45]. The crosslinking density can be varied by modifying the ratio of crosslinking units to polymer backbone units during polymer- and network formation or by the extent of the crosslinking reaction performed after the film formation. The state of the polymer when the crosslinks are introduced is another important aspect of the crosslinking process. The difference between dry crosslinking (in photocrosslinking of a neat polymer film) and wet crosslinking (in crosslinking polymerization in water) becomes evident when the polymer layer is attached to a substrate surface. The latter case of crosslinking under equilibrium conditions does not lead to strain when the swollen network is anchored to the substrate, while the situation is different for the neat polymer being crosslinked and surface-attached before swelling. The anchoring restricts movement and swelling at the hydrogel-substrate interface and allows network expansion in a highly anisotropic fashion in only one direction, away from the surface [30,33,41,85]. A consequence of this surface confinement in hydrogel layers is a reduced swelling compared to the unrestricted 3D hydrogel by a factor of around 5 to 10 [30,38]. 

The swelling kinetics is another characteristic property of hydrogel films that strongly depends on structural parameters, such as chemical composition of the polymer backbone and morphological structure of the hydrogel material. For instance, incorporating ionic groups such as sodium acrylate into a poly(*N*-isoproylacrylamide)-based hydrogel network was shown to increase the swelling speed by orders of magnitude in bulk gels [84]. The incorporation of the ionic groups increases the hydrophilicity of the hydrogel material and at the same time the acrylate moieties potentially phase separate from the hydrophobic polymer segments into highly polar regions upon the thermally induced collapse. These regions might act as hydrophilic channels that allow water transport even in the compact collapsed state. Besides the chemical composition, the morphological structure of the hydrogel material can have a strong influence on the swelling behavior [83]. Structural features like pores and surface-to-volume ratio directly influence the water transport, as the swelling and collapse processes are determined by the diffusion process of water in and out of the network. An increase of the swelling kinetics due to the presence of large interconnected pores was found for poly(acrylamide-*co*-acrylic acid) hydrogels [86]. Moreover, it was reported that microstructured hydrogel films were swelling much faster than macroscopic objects, due to the small gel dimensions and the sponge-like nanostructure of the hydrogel material [45]. A detailed study of the swelling kinetics revealed a two-stage process in zwitterionic hydrogel films, an initial fast diffusion followed by a subsequent slower process controlled by the relaxation of polymer fragments [29]. A similar two-stage kinetics was found in plasma crosslinked thermoresponsive hydrogel films [47]. Within minutes a fast dynamic swelling took place, which was followed by a slow equilibrium swelling over several days.

A number of methods such as ellipsometry [29,30,47], SPR and SPR/OWS [33,35,44], neutron reflectivity [48], z-scans in confocal fluorescence microscopy [78], and AFM [39,42,81] have been employed to measure the thickness of hydrogel films. These methods allow the determination of the swelling ratio between the swollen and the dry or collapsed film thickness, and often also provide information about the swelling kinetics. The variation of the mechanical properties during swelling can also be measured by AFM and quartz crystal microbalance [39,42,81,87,88,89].

Responsive hydrogels can abruptly change their physico-chemical properties in response to external stimuli, such as temperature, pressure, light, electric- or magnetic fields, solvent composition, pH or salt concentration. The response of the hydrogel to the external stimuli can be continuous, due to a gradual change of the environmental parameter, or it can be discontinuous in the form of an abrupt volume change when passing a critical value of the external parameter. The LCST is one of the most exploited discontinuous responses in hydrogels. The phase transition temperature is intrinsically linked to the chemical nature of the polymer backbone and can be tuned by the chemical composition of the polymer. It was shown that for thin films of a copolymer composed of *N*-isopropylacrylamide with 2-(dimethylmaleimido)-*N*-ethylacrylamide the LCST was lowered, while *N*,*N-*dimethylacrylamide raised the LCST [44]. For a series of photocrosslinked hydrogel layers based on poly(*N*-isopropylacrylamide) with identical chemical structure and film thickness, but with different crosslinking densities, it was found that the transition temperature dropped with increasing the crosslink density (induced by prolonged irradiation times, but not chemical composition) [42]. Light is another stimulus that has been employed to induce changes in the polymer conformation and swelling state in solution and 3D gels by modification with chromophores such as azobenzenes and triphenylmethane leuconitrile moieties [12]. In addition, electro-sensitivity was reported for surface-attached charged peptides with a bioactive entity incorporated at the end which can undergo a conformational switching upon application of a surface potential leading to the reversible exposure or concealing of the bioactive moiety [13]. The same concept may be applied to surface-attached hydrogel films for regulating biomolecular interactions by irradiation with light.

The nature of the swelling solvent is a purely chemical stimulus of substantial relevance. For example, the effect of variation of the solvent composition in water-ethanol mixtures on the transition temperature was studied in thin poly(*N*-isopropylacrylamide) films by SPR/OWS [34]. It was observed that the addition of small amounts of ethanol to the water phase dramatically lowers the transition temperature in the hydrogel film, due to a cononsolvency effect. With pure ethanol solutions no volume phase transition is observed. Furthermore, solutes in the aqueous phase are chemical stimuli that can influence the hydrogel swelling state by interaction with the polymer. For example, pH changes in ionic hydrogels can substantially alter the ionization of charged groups and influence the electrostatic interactions in the polymer, while the addition of salt affects both the electrostatic interactions by charge screening and the osmotic pressure due to the introduction of solvated ions. The effect of salt (NaCl) concentration on the layer thickness and homogeneity in hydrogel films composed of poly(*N*-isopropylacrylamide) copolymers with COOH groups was investigated by SPR/OWS. It was found that a slight increase of the layer thickness and inhomogeneities was evident with increasing salt concentration up to a critical concentration of about 1 M, above which the gel collapsed [35,80].

## 4. Biosensor Implementations

In this section, an overview of implementations of evanescent wave optical biosensors with hydrogel binding matrix is presented. Representative examples of biosensor schemes relying on molecular imprinted hydrogels, responsive gels modified with enzymes, and biosensors taking advantage of nucleic acids and immunoassays are introduced and their performance characteristics are discussed. Let us note that this section does not provide a complete review of this topic. Rather, interesting approaches were selected for the sake of illustration as summarized in Table 1. The scope of this discussion is limited to biosensors with crosslinked polymer networks attached to their surface while polymer brushes (thinner structures in which polymer chains are individually anchored to a surface) are not included.

**Table 1:** Overview of hydrogel biosensors based on evanescent wave optics.

Recognition element	Material	Detection method	Thickness(d_h_)	Analyte	LOD(sample)	Ref.
MIP	poly(2-vinylpyridine)-AuNPs	LSPR	31 nm	cholesterol	n.a. (chloroform)	[90]
	acrylamidophenylboronic acid- acrylamide	SPR	22 nm	NAD(P)^+^ NAD(P)H	1 µM (buffer)	[91]
	acrylic acid-	SPR	~6 µm (dry)	dopamine	1 nM (water)	[92]
	*N*-isopropylacrylamide-					
	*N,N*’-methylenbisacrylamide-AuNPs					
	poly(*N*-(*N*-propyl)acrylamide)	SPR	~300 nm	Theophylline	10 µM (buffer)	[93]
	methacylic acid-ethylene	SPR	251 nm	atrazine	5 pM (acetonitrile)	[94]
	glycol dimethacrylate-AuNPs					
Enzyme	agaros-guar gum	OWS	12 µm	sucrose	25 pM (buffer)	[95]
	polysaccharide					
	agarose co-polymer	OWS	1 µm	paraoxon	6 nM (buffer)	[96]
	agarose co-polymer	OWS-Fluorescence	1 µm	glucose	3 µM (buffer)	[96]
	alginate-gelatin-AgNPs	LSPR	20 nm (dry)	glucose	0.1 m (buffer)	[97]]
	acrylamide-bisacrylamide-AgNPs	LSPR	~1 mm	glucose	10 pM (buffer)	[98]
Nucleic acid	POWT	SPR	8 nm(dry)	DNA	n.a. (buffer)	[99]
	aptamer-polyacrylamide	LSPR	n.a.	adenosine	n.a. (buffer)	[100]
	aptamer-polyacrylamide	LSPR	n.a.	cocanie	n.a. (buffer)	[101]
Immuno-assay	carboxymethyl dextran	LR-SPR	~1 µm	f-PSA	0.68 n (buffer)	[23]
	carboxymethyl dextran	LRSP-FS	~1 µm	f-PSA	34 fM (buffer)	[23]
					330 fM (human serum)	
	poly(*N*-isopropylacrylamide)	HOWS	~2 µm	IgG	10 pM(buffer)	[102]
	poly(ethyleneglycol) methacrylate-2-hydroxyethyl methacrylate	SPRi	5–45 nm	HSA and calmodulin	n.a.(buffer)	[25]

### 4.1. Molecular Imprinted Hydrogel-Based Biosensors

**Figure 6:**
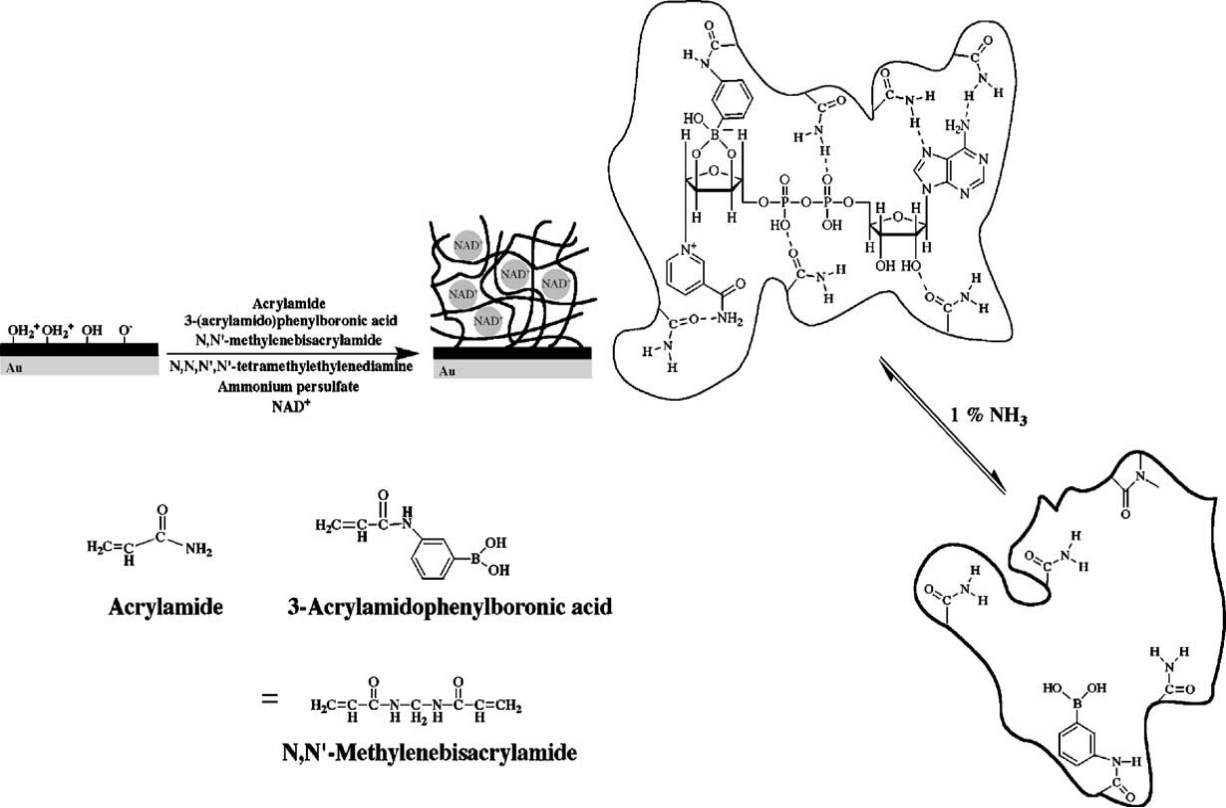
NAD(P)+/NAD(P)H cofactors-imprinted hydrogel with an acrylamide-acrylamidophenylboronic acid copolymer on an Au-coated glass for SPR-based detection. (Reproduced with permission from [91]).

Molecular imprinted hydrogels have attracted a great deal of attention over the last decade due to their potential to provide robust means for recognition of target analytes that is stable over long time [103,104]. In biosensors, we witnessed numerous implementations of such materials for direct refractometric-based detection of low molecular weight analytes relevant to medical diagnostics and environmental monitoring. In these systems, the specific capture of target analyte in the hydrogel with imprinted moieties alters its density and refractive index, which is subsequently optically detected by methods including surface plasmon resonance (SPR) or optical waveguide spectroscopy (OWS). Hydrogel-based molecular imprinted matrices with responsive properties were shown to amplify the detected optical changes through additional binding-induced collapse or swelling. A molecular-imprinted hydrogel with an acrylamide-acrylamidophenylboronic acid copolymer was developed for the detection of β-nicotinamide adenine dinucleotide (NAD^+^), β-nicotinamide adenine dinucleotide phosphate (NADP^+^), and their reduced forms (NADH and NADPH) [91]. This material is shown in Figure 6 and the binding of target molecules triggered its swelling. In combination with SPR readout detection of NAD(P)^+^ and NAD(P)H in the concentration range of 1 µM to 1 mM was reported. In order to provide stronger binding-induced refractive index changes, gold nanoparticles (AuNPs) were incorporated into molecular imprinted hydrogels [92,105]. This approach offered enhanced sensor response upon the swelling and collapse of a gel through the distance-dependent interaction between AuNPs embedded in the gel. For instance, an SPR sensor for detection of dopamine, which is an important neurotransmitter, was reported to utilize this principle by using imprinted acrylic acid, *N*-isopropylacrylamide, and *N,N*’-methylenbisacrylamide hydrogel [92]. 

Besides monolithic molecular imprinted hydrogel films, microgels were used for a construction of a binding matrix which offers faster diffusion of analyte to the polymer. Poly(*N*-(*N*-propyl)acrylamide) microgel particles with a diameter of 300 nm were imprinted by theophylline and attached to a gold surface [93]. The binding of target theophylline molecules increased the hydrophilicity of the microgel particles, which resulted in their swelling. The associated decrease in the refractive index due to the theophylline capture was subsequently detected by SPR. The sensor was capable of specific detection of theophilline at concentrations above 10 μM.

### 4.2. Enzyme-Based Biosensors

Employing of enzyme recognition elements offers a possibility for detection of numerous analytes important for medical diagnostics. The interaction of an enzyme with respective target analyte typically triggers a catalytic reaction, which changes the characteristics of the local environment in the hydrogel (e.g., pH). When the enzyme is immobilized in a hydrogel responsive to these changes, these variations can be translated to swelling or collapse of the gel and subsequently converted to an optical output of the sensor.

A sucrose biosensor was developed based on an agarose guar gum-based binding matrix with incorporated acid invertase and glucose oxidase [95]. This film with a thickness of 12 μm was attached to a single mode optical waveguide that probed the refractive index changes ascribed to the specific reaction of sucrose in the matrix. Monitoring of the waveguide attenuation allowed rapid detection of sucrose in 110 s with the limit of detection as low as 25 pM. In another study, a waveguide structure with a 1 μm thick sol-gel matrix was employed for the absorption and fluorescence spectroscopy-based detection of glucose [96]. The sol-gel matrix was modified with glucose oxidase and a fluorescent ruthenium complex, which is sensitive to pH. The sensing principle was based on the quenching of ruthenium complex upon the release of gluconic acid that was accompanied by bio-oxidation of glucose and allowed detecting of glucose concentrations above 3 µM.

**Figure 7:**
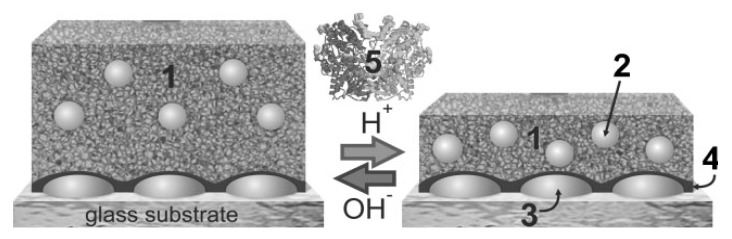
Ultrathin alginate-gelatin gel film (1) with silver nanoparticles (2) immobilized on silver nanoislands (3) via a poly(glycidyl methacrylate) layer (4). The film undergoes a reversible swelling transition in response to pH changes caused by the biocatalytic reaction of GOx (5) and glucose (Reproduced with permission from [97]).

Various biosensors based on “smart” hydrogel/nanoparticles composites and enzyme reactions were reported [97,106]. This approach employed the molecular binding-induced swelling, which modulated the (distance-dependent) interaction between metallic nanoparticles incorporated in a gel (see Figure 7). The swelling or collapse of the hydrogel host altered the nanoparticle interaction strength and lead to a shift in the localized surface plasmon resonance (LSPR). For instance, a 20–25 nm thick pH-responsive alginate-gelatin film with encapsulated silver nanoparticles was deposited on the surface of a solid support with silver islands. Through biocatalytic reaction of glucose oxidase (GOx) in the gel, the detection of glucose at concentrations down to 0.1 mM was reported.

In another work, silver nanoparticles were coated with a responsive hydrogel (composed of acrylamide, bisacrylamide and polyvinylpyrrolidone) in order to function in a glucose biosensor. The sensing mechanism was based on the formation of a reduced flavin adenine dinucleotide (FAD) anion, gluconic acid and hydrogen peroxide upon glucose turnover that resulted in the swelling of hydrogel and degradation of silver nanoparticles [98]. Detection of glucose at as low as 10 pM concentration was demonstrated.

### 4.3. Nucleic Acids-Based Biosensors

**Figure 8:**
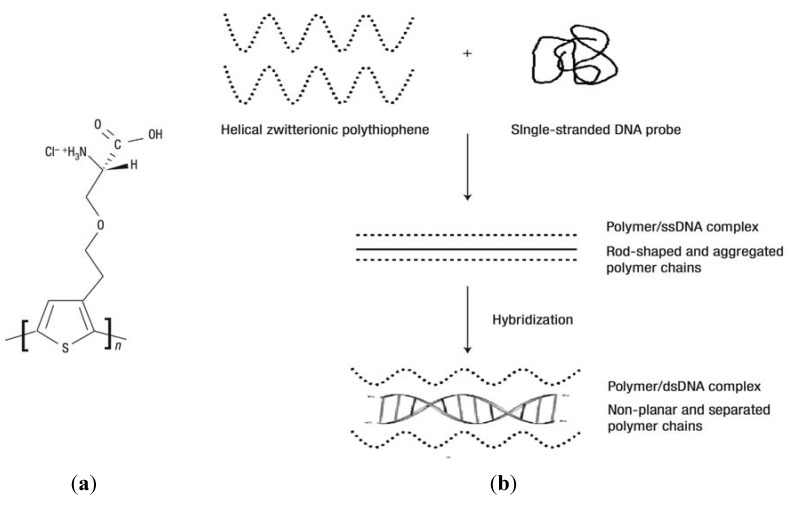
(**a**) The repeat unit of poly(3-[(S)-5-amino-5-carboxyl-3-oxapentyl]-2,5-thiophenylene hydrochloride) (POWT) and (**b**) formation of a POWT/DNA probe complex. Geometrical changes of POWT (dotted lines) upon hybridization with ssDNA (solid lines) and formation of the double-stranded, helical DNA structure (dDNA). (Reproduced with permission from [107]).

Hydrogel matrices carrying oligonucleotide probes were implemented for the detection of nucleic acids by using SPR and fluorescence spectroscopy. For instance, a water-soluble polythiophene polymer composed of poly(3-[(S)-5-amino-5-carboxyl-3-oxapentyl]-2,5-thiophenylene hydrochloride) (POWT) with zwitterionic peptide-like side chains was used for detection of single stranded DNA [99,107]. As Figure 8a shows, the zwitterionic side-chains carry serine carboxylic groups with a p*K_a_* of 2.19 and an amino group with a p*K_a_* of 9.21, which are responsible for pH-dependent conformational transitions in the gel. POWT undergoes aggregation accompanied by a transition to a denser and straightened structure (rod-shaped polymer backbone) upon the binding of target ssDNA to DNA probes immobilized within the gel via electrostatic and hydrogen bonding. Refractive index changes associated with these conformational changes were translated to a sensor signal by SPR. In addition, the POWT exhibits fluorescence characteristics sensitive to electrostatic interaction of polymer chains. It was shown that the emission band of POWT polymer shifts to longer wavelengths as the net charge of the polymer side-chains becomes more negative [108]. A negatively charged probe DNA couples with polymer chains via hydrogen bonding and its hybridization with target ssDNA is accompanied with a disruption of this interaction, see Figure 8b. Therefore, upon the capture of target ssDNA the intensity of the emitted fluorescence light is increased and its wavelength is blue shifted [107]. This method allowed detection of DNA at a concentration as low as 6.7 nM.

Another type of nucleic acid biosensor, based on an aptamer-crosslinked polyacrylamide hydrogel, was developed for detection of small molecules such as adenosine [100] and cocaine [101]. This hydrogel was modified by two DNA strands that were coupled by an aptamer linker and thus contributing to hydrogel crosslinking. The binding of the target to the aptamer resulted in a disruption of the crosslinking associated with a gel dissolution and subsequent release of trapped reporter AuNPs or enzyme. The released AuNPs or the enzyme activity was monitored by absorption spectroscopy in the visible part of the spectrum and allowed detection of cocaine at concentrations below 1 μM within 10 min [101].

### 4.4. Immunoassay-Based Biosensors

Immunoassays represent an established method for detection of chemical and biological analytes owing to commercial availability of antibodies against a wide range of analytes, their versatility and high affinity. In biosensors with hydrogel binding matrix, antibodies are immobilized in a hydrogel film, which exhibits a huge binding capacity and highly open network structure through which molecular analytes can rapidly diffuse. These materials offer the advantage of an increased binding capacity and decreased steric hindrance with respect to more traditional monolayer architectures. Three examples of biosensors developed for immunoassays-based detection of target molecules by label-free techniques relying on monitoring a binding-induced refractive index changes and fluorescence spectroscopy, as discussed in the following section.

A biosensor based on surface plasmon resonance imaging (SPRi) employed a hydrogel matrix composed of poly(ethylene glycol) methacrylate and 2-hydroxyethyl methacrylate with a lateral gradient [25]. Biospecific interaction of serum albumin (HSA)/anti-HSA antibody and calmodulin (CaM)/calmodulin binding domain (CBD) affinity pairs was studied as a function of thickness and composition of the matrix. CaM with low molecular weight was shown to diffuse and interact throughout the entire hydrogel matrix. However, diffusion of larger HSA was hindered by the low porosity of hydrogel matrix and thus it reacted only on the gel surface. The sensitivity for detection of CaM was dependent on the thickness of the hydrogel, and the highest detection sensitivity for this analyte was achieved for a hydrogel matrix thickness of 70 nm.

**Figure 9:**
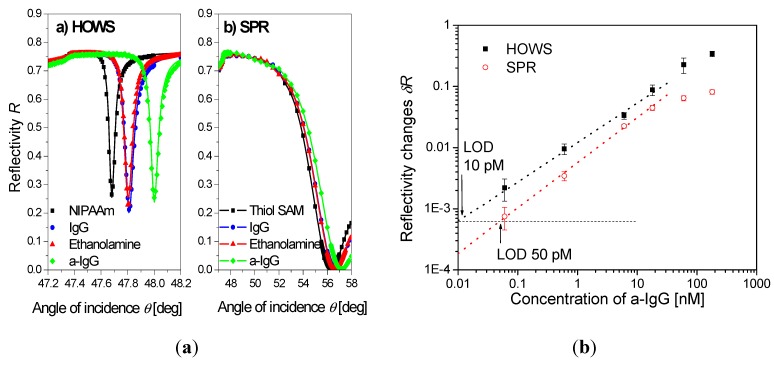
(**a**) Reflectivity spectra measured by hydrogel optical waveguide spectroscopy (HOWS) in an approximately 2 μm thick hydrogel binding matrix, compared with those for regular SPR with thiol SAM surface architecture. The spectra of the pristine hydrogel (squares), after immobilization of IgG capture antibodies (circles), passivation with ethanolamine (triangles), and affinity capture of the a-IgG target analyte (diamonds) are shown. (**b**) Calibration curve obtained by HOWS and SPR for a model immunoassay experiment. (Reproduced with permission from [102]).

A highly swollen poly(*N*-isopropylacrylamide) hydrogel with a thickness around 2 μm was shown to simultaneously serve as a large binding capacity matrix and a waveguide in a label-free biosensor utilizing hydrogel optical waveguide spectroscopy (HOWS) [102]. Owing to the low polymer volume content of f~0.1 even larger molecules, such as IgG, diffused rapidly into the gel and reacted with the immobilized biomolecular recognition elements. In comparison with conventional SPR utilizing a monolayer surface architecture, the HOWS detection method provided an order of magnitude better resolution in the refractive index measurements due to the narrower resonance associated with the excitation of hydrogel waveguide modes (see Figure 9a). In a model immunoassay experiment, HOWS offered a 5-fold lower limit of detection for IgG molecules with respect to the regular SPR method, as illustrated by the calibration curve in Figure 9b. Larger sensitivity enhancements are expected for detection of smaller molecules that diffuse faster into the gel and for which the HOWS biosensor scheme can take full advantage of its large binding capacity.

**Figure 10:**
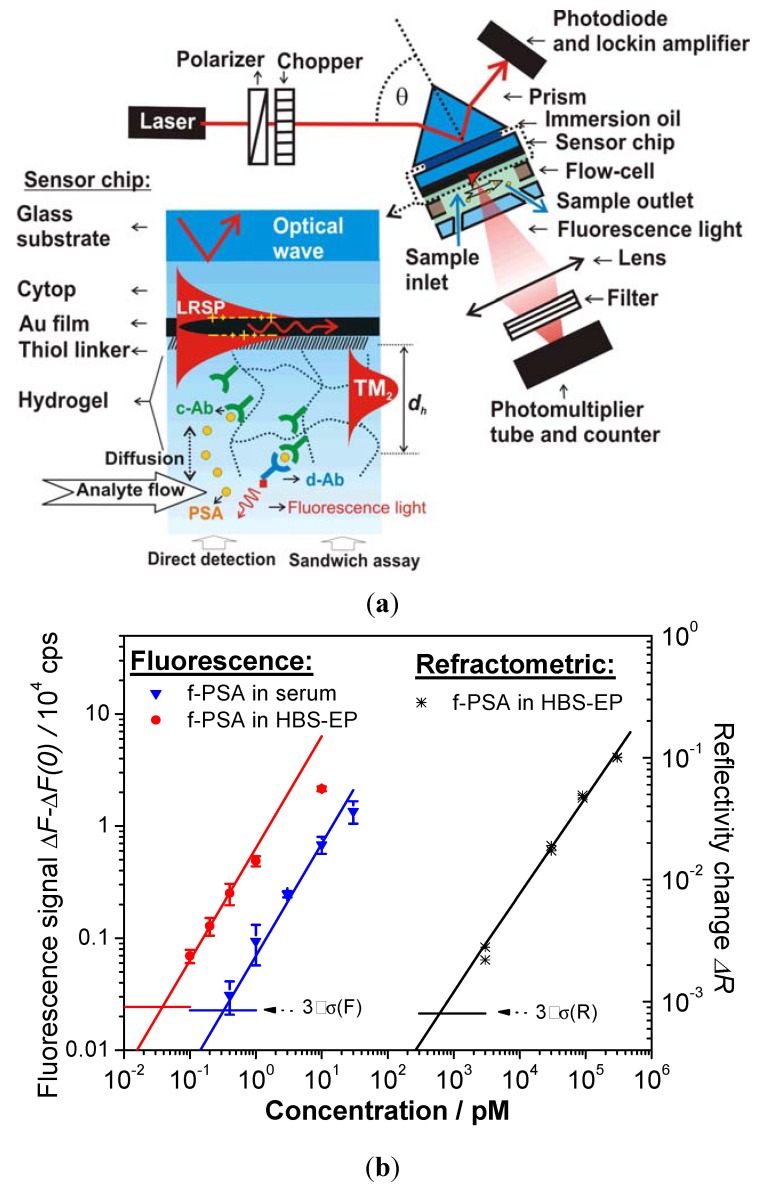
(**a**) Optical setup for the excitation of long range surface plasmons (LRSPs) on the sensor surface with photo-crosslinked carboxymethyl dextran hydrogel binding matrix for sandwich immunoassay detection of f-PSA. (**b**) Calibration curves measured for direct and fluorescence-based detection of f-PSA in a buffer (HBS-EP) and human serum (Reproduced with permission from [23]).

In another immunosensor, detection of free prostate specific antigen (f-PSA) was carried out by using a highly swollen, 1 μm thick hydrogel binding matrix of photo-crosslinked carboxymethyl dextran (see Figure 10a). In this work, a sandwich immunoassay was combined with long range surface plasmon (LRSP) field-enhanced fluorescence spectroscopy [23]. The strong electromagnetic field intensity, provided by the excitation of LRSP modes, enhanced the excitation rate of chromophore-labeled secondary antibodies, which were bound to previously captured f-PSA in the hydrogel. This process directly translated to an increased fluorescence signal. As seen in the calibration curve in Figure 10b, a low femtomolar limit of detection was achieved for LRSP-enhanced fluorescence spectroscopy. This limit of detection was about 4 orders of magnitude lower than that observed for direct detection of f-PSA (through measuring refractive index changes of the gel by LRSP spectroscopy). In addition, the performance of the fluorescence-based readout was less affected by nonspecific sorption and even allowed the analysis of f-PSA in human serum. 

## 5. Conclusions

Hydrogel materials possess many properties that endow them with attractive features for a wide range of applications. Specifically, the thin-film format of hydrogels attached to solid surfaces is of great technological interest, as it represents a “smart” coating that can be microstructured and integrated into microfabrication processes. As a consequence of the surface attachment, the hydrogel swells only in one dimension and, due to the large surface-to-volume ratio in the thin film compared to bulk gels, water and solute diffusion in and out of the hydrogel as well as response times are highly accelerated. In important areas such as medical diagnostics and environmental monitoring, hydrogel materials allowed impressive advances in biosensor performance and helped to push these technologies towards applications. Particularly, we witnessed development of new hydrogel materials with improvements in their recognition characteristics through incorporation of specific moieties by molecular imprinting or coupling of biomolecular recognition elements. We expect that future biosensor technologies will increasingly take advantage of “smart” hydrogels with responsive properties, which will increase their sensitivity and allow implementation of new simplified detection schemes. In conjunction with optical biosensors, advances in molecular imprinted large binding capacity-materials hold potential to deliver urgently needed tools for direct label-free detection of low molecular weight molecules such as drugs in medical diagnostics or pollutants in environmental monitoring. For such analytes, currently established technologies including immunoassay-based SPR lack the sensitivity and long-term stability, which needs to be addressed in future research.
